# Placental structural adaptation to maternal physical activity and sedentary behavior: findings of the DALI lifestyle study

**DOI:** 10.1093/humrep/deae090

**Published:** 2024-05-10

**Authors:** Saghi Zafaranieh, Monika Siwetz, Barbara Leopold-Posch, Daniel Kummer, Berthold Huppertz, Gernot Desoye, Mireille van Poppel, DALI Core Investigator Group, Gernot Desoye, David Simmons, Rosa Corcoy, Juan M Adelantado Perez, Alexandra Kautzky-Willer, Jürgen Harreiter, Peter Damm, Elizabeth Mathiesen, Dorte M Jensen, Lise Lotte T Andersen, Fidelma Dunne, Annunziata Lapolla, Maria G Dalfra, Alessandra Bertolotto, Mireille van Poppel, Judith G M Jelsma, Sander Galjaard, Ewa Wender-Oegowska, Agnieszka Zawiejska, David J Hill, Roland Devlieger, Frank J Snoek

**Affiliations:** Department of Human Movement Science, Sport and Health, University of Graz, Graz, Austria; Division of Cell Biology, Histology and Embryology, Gottfried Schatz Research Center, Medical University of Graz, Graz, Austria; Department of Obstetrics and Gynecology, Medical University of Graz, Graz, Austria; Division of Cell Biology, Histology and Embryology, Gottfried Schatz Research Center, Medical University of Graz, Graz, Austria; Division of Cell Biology, Histology and Embryology, Gottfried Schatz Research Center, Medical University of Graz, Graz, Austria; Department of Obstetrics and Gynecology, Medical University of Graz, Graz, Austria; Department of Human Movement Science, Sport and Health, University of Graz, Graz, Austria

**Keywords:** maternal obesity, physical activity, sedentary behavior, fetal-placental circulation, physiological adaptation, placenta, angiogenesis

## Abstract

**STUDY QUESTION:**

Are maternal levels of moderate-to-vigorous physical activity (MVPA) and sedentary time (ST) in obese pregnant women associated with placental structural adaptations for facilitating oxygen delivery to the fetus?

**SUMMARY ANSWER:**

Higher maternal MVPA and ST are associated with a higher density of villi, a proxy measure of placental surface area for oxygen delivery to the fetus, without further added placental vessels.

**WHAT IS KNOWN ALREADY:**

Physical activity during pregnancy intermittently reduces uterine blood flow, potentially limiting placental and fetal oxygen supply. The placenta can mount several adaptive responses, including enlargement of the surface area of villi and/or feto-placental vessels to accommodate fetal needs. Early research on the morphology and growth of the placenta with exercise interventions has shown inconsistencies and is lacking, particularly in non-lean pregnant women.

**STUDY DESIGN, SIZE, DURATION:**

This study is a secondary longitudinal analysis of the vitamin D and lifestyle intervention for gestational diabetes prevention (DALI) randomized controlled trial. The prospective study was conducted between 2012 and 2015 in nine European countries at 11 different sites. In this analysis, 92 pregnant women with a BMI ≥ 29 kg/m^2^ were combined into one cohort.

**PARTICIPANTS/MATERIALS, SETTING, METHODS:**

MVPA and percentage of time spent sedentary (% ST) were measured with accelerometers during gestation. Placental sections were immunostained for endothelial cell-specific CD34. Artificial intelligence (AI)-based stereology assessed villous density, number, and cross-sectional area of vessels on whole-slide images and in selected regions comprising peripheral villi only, where the majority of vascular adaptations occur. Expression of pro- and anti-angiogenic factors was quantified using molecular counting analysis.

**MAIN RESULTS AND THE ROLE OF CHANCE:**

In multivariable regression, higher levels of maternal MVPA (min/day) were associated with a higher density of villi in both whole-slide images (beta 0.12; 95% CI 0.05, 0.2) and selected regions (0.17; CI 0.07, 0.26). Unexpectedly, ST was also positively associated with density of villi (0.23; CI 0.04, 0.43). MVPA and ST were not associated with vessel count/mm^2^ villous area, vessel area, or pro- and anti-angiogenic factor mRNA expression. All estimates and statistical significance of the sensitivity analyses excluding smokers, women who developed gestational diabetes or pre-eclampsia and/or pregnancy-induced hypertension were similar in the main analysis.

**LIMITATIONS, REASONS FOR CAUTION:**

The placenta is a complex organ undergoing dynamic changes. While various adjustments were made to account for different maternal contributing factors, in addition to the outcome measures, various other factors could impact oxygen delivery to the fetus.

**WIDER IMPLICATIONS OF THE FINDINGS:**

For the first time, we evaluated the association between placental structures quantified using an AI-based approach with objectively measured physical activity and ST at multiple time points in pregnant women with obesity. The observed adaptations contribute to the advancement of our understanding of the hemodynamics and adaptations of the placental unit in response to MVPA and ST. However, our results might not be generalizable to lean pregnant women.

**STUDY FUNDING/COMPETING INTEREST(S):**

The DALI project has received funding from the European Community’s 7th Framework Program (FP7/2007–2013) under grant agreement no. 242187. The funders had no role in study design, collection of data, analyses, writing of the article, or the decision to submit it for publication. The authors have no conflicts of interest to declare.

**TRIAL REGISTRATION NUMBER:**

ISRCTN70595832.

## Introduction

The placenta as the interface between the pregnant woman and her fetus mediates maternal exposures to the fetus, supplies it with oxygen and nutrients, and supports its growth and development (Desoye *et al.*, 2011; Huppertz, 2023a). The key structures of the placenta, placental villi, are the primary tissue accounting for placental oxygen and nutrient uptake (Lewis *et al.*, 2013). During the first half of pregnancy, the expansion of the villous epithelium, the villous trophoblast together with the villous vascular network results in a massive increase in size and volume of placental villi. In the second half of pregnancy, villous growth, and especially the development of peripheral villi is mainly caused by angiogenesis and growth of vessels (Kingdom *et al.*, 2000; Huppertz and Peeters, 2005; Huppertz, 2023b). Intra-placental and fetal oxygen levels as well as paracrine factors secreted by villous cytotrophoblasts and placental macrophages (Hofbauer cells) determine the degree and type of feto-placental angiogenesis (Demir *et al.*, 2006; Loegl *et al.*, 2017; Desoye and Carter, 2022).

A range of mechanisms has evolved to adapt placental structure and function to accommodate fetal needs. An increased maternal metabolic burden such as in obesity, however, has the potential to compromise these adaptive effects (Desoye and Wells, 2021). Maternal obesity increases the generation of free radicals, as well as inflammation in the intrauterine environment. The placenta shows signs of altered development in obesity (Mele *et al.*, 2014; Stuart *et al.*, 2018; Hoch *et al.*, 2019). These placental changes include alterations of vascularization that result in the adaptation of blood flow to the fetus to accommodate its increased oxygen demand as a result of its increased oxidative metabolism (Chen *et al.*, 2019; de Barros Mucci *et al.*, 2020; Nogues *et al.*, 2021; Desoye and Carter, 2022).

Physical activity (PA) has various beneficial effects on various organ systems, including skeletal muscle and adipose tissue, and regulates carbohydrate metabolism (Mul *et al.*, 2015; Stanford and Goodyear, 2016; Thyfault and Bergouignan, 2020). Also, during an uncomplicated pregnancy, PA has numerous health benefits for a woman and her offspring (Simmons *et al.*, 2017; Dipietro *et al.*, 2019; Zheng *et al.*, 2020), such as reduction of excessive gestational weight gain and gestational diabetes or reduced oxidative stress and inflammation (Bogaerts *et al.*, 2012; Thangaratinam *et al.*, 2012; Shepherd *et al.*, 2017; Ferrari and Joisten, 2021; Zafaranieh *et al.*, 2023b). However, high-intensity PA might decrease oxygen and nutrient delivery to the placenta, and subsequently to the fetus, by diverting blood to skeletal muscles of the pregnant woman and lowering blood and, hence, oxygen supply of the placenta (Hart *et al.*, 1956; Rowell, 1974; Lotgering *et al.*, 1983; Clapp, 2003; Clapp and Catherine, 2012; Salvesen *et al.*, 2012; Szymanski and Satin, 2012; Nguyen *et al.*, 2013). In a small cohort of pregnant women engaged in weight-bearing aerobic exercise, and using the point counting stereological method, villous volume, especially of terminal villi, was greater in the exercise group (Clapp *et al.*, 2000a). This was found in lean women and was not further investigated in larger or other populations and settings, such as in women with obesity.

In pregnancies complicated by abnormal maternal metabolic or endocrine status, the placenta may undergo compensatory changes in its structure and function to maintain adequate oxygen supply during periods of fetal growth or in response to transient placental and fetal oxygen deficit (Sandovici *et al.*, 2012; Desoye and Wells, 2021).

Considering the adaptability of the placenta and the intimate ties between its development and fetal oxygenation, we hypothesized that moderate-to-vigorous physical activity (MVPA) and sedentary time (ST) among women with obesity associate with structural adaptations of the placenta to facilitate adequate oxygen delivery. To investigate this hypothesis, we tested the association between objectively measured PA and ST during pregnancy and structural changes in the placenta of obese women. We have focused on two aspects of placental structure, i.e. villous area (as a measure for growth) and number of villous vessels (as a measure for vascularization) using automated stereological quantification.

## Materials and methods

### Participants

This is a secondary analysis of the vitamin D and lifestyle intervention for gestational diabetes mellitus (GDM) prevention (DALI) study, a multicenter randomized controlled trial conducted between 2012 and 2015 in nine European countries at 11 different sites (Austria, Belgium, Denmark (Odense, Copenhagen), Ireland, Italy (Padua, Pisa), Netherlands, Poland, Spain and UK). The DALI Lifestyle Study was designed to evaluate the effects of healthy lifestyle counselling on GDM progression in obese pregnant women (Jelsma *et al.*, 2013).

Participants of the study were pregnant women with a singleton pregnancy, gestational age of less than 20 weeks, aged ≥18 years, and with a pre-pregnancy BMI of ≥29 kg/m^2^. At baseline, women who were diagnosed with GDM based on International Association of Diabetes and Pregnancy Study Group (IADPSG) criteria (International Association of Diabetes and Pregnancy Study Groups Consensus Panel; Metzger *et al.*, 2010), or had a pre-diagnosis of diabetes, chronic medical conditions, psychiatric disorders, inability to walk 100 m safely, inability to communicate effectively with their lifestyle coach due to language proficiency limitations or required a complex diet were excluded from the study.

After giving written informed consent, women were randomized into four groups receiving counselling for healthy eating (HE), physical activity (PA), healthy eating + physical activity (HE + PA), and a control group receiving usual care (UC) in the lifestyle trial. For this analysis, participants were combined into one cohort to assess the data longitudinally, to control for potential confounding variables, and to enhance the statistical power of analysis by including the PA levels of a larger sample size.

### Ethical approval

The DALI study was approved by all local ethics committees and was registered under trial registration number ISRCTN70595832. The present sub-study was additionally approved by the ethics committee of the Medical University of Graz (number 30-485 ex17/18).

### Data collection

Data on anthropometrics, blood samples and questionnaires were collected at four time periods (<20 weeks, 24–28 weeks, and 35–37 weeks and at delivery). The questionnaire included information on age, pre-pregnancy weight, ethnicity, parity, smoking status, alcohol consumption, and medical history. Data on birth outcomes were collected from medical files.

PA was measured objectively by accelerometer (ActiGraph GT1m, GT3X+ or Actitrainer; ActiGraph, Pensacola, FL, USA) at <20 weeks, 24–28 weeks, and 35–37 weeks. Women were asked to wear the accelerometer over their right hip for at least 3 days at each time period and remove it only when swimming or showering and to document the reason and the duration of the removal. The average time spent sedentary (<100 counts/min), in light (100–1951 counts/min), and in MVPA (>1951 counts/min) was determined using Freedson cut-off points. Additionally, the amount of time spent swimming was incorporated into the calculation of minutes spent in MVPA. ST was calculated as a proportion of overall daily accelerometer wear time (% ST) for analysis purposes. Since light physical activity, together with ST and MVPA, makes up all of the measured daily physical activity, it was left out from the model due to multicollinearity.

### Placental tissue collection and histological examination

Placentas were collected within 30 min after delivery. Per placenta, from each of the quadrants, one piece was dissected comprising the maternal and fetal side and stored at −20°C in cryotubes filled with RNA-later (Thermo Fisher Inc, Vienna, Austria)until analysis. For this study, we chose one piece of the fetal side from one quadrant, and from the opposite quadrant one piece of the maternal side. RNA-later was removed and the samples were fixed in 10% formalin overnight. After dehydration and paraffin infiltration in a series of alcohol solutions, beginning with 60% and progressing to 100% alcohol and Histolab Clear (Histolab, Askim, Sweden) in an Excelsior AS Tissue Processor (Thermo Shandon Limited, Runcorn, UK), samples were embedded into paraffin blocks.

A Thermo Scientific HM 355S rotary microtome (Thermo Fisher Scientific, Walldorf, Germany) was used for paraffin sectioning. Sections (5 µm) were mounted on Superfrost PlusSlides (Epredia, USA) and dried on the heating plate for approximately 12 h at 40°C. Before deparaffinization, the sections were baked for 20–30 min at 60 °C on the heating plate.

Before conducting immunohistochemistry, the slides were subjected to deparaffinization and rehydration. Antigen retrieval was performed by heating the slides in Tris-EDTA Buffer (pH 9.0) using a microwave for two rounds of 20 min each at 150 W. Subsequently, the slides were allowed to cool for 20 min at room temperature and the standard immunohistochemistry method was performed using the UltraVision Large Volume Detection System HRP Polymer Kit (Epredia, USA) according to the manufacturer’s protocol (Shi *et al.*, 1999). In brief, after blocking endogenous peroxidase for 10 min with hydrogen peroxidase block, slides were washed three times with Tris-buffered saline including 0.05% Tween 20 (TBS-T). Following background blocking with Ultra Vision Protein Block for 5 min, sections were then incubated with CD34 antibody (Dako, Agilent Technologies, Denmark; Monoclonal Mouse, Clone QBEnd 10) with a concentration of 12 mg/l and diluted 1:400 in Antibody Diluent (Agilent, Santa Clara, CA, USA). Slides were additionally incubated with primary anti-mouse antibody Enhancer for 10 min. Subsequently, slides were washed three times and were incubated with Large Volume HRP Polymer for 15 min at room temperature. After another three steps of washing with TBS-T, slides were incubated with the substrate 3-amino-9-ethylcarbacole (AEC, Chromogen Single Solution; Abcam, UK) for 10 min. Subsequently, they underwent three rounds of washing with distilled water, and the nuclei were stained with Hemalaun. Finally, the slides were mounted using Kaiser’s Glycerin Gelatine (Merck, Darmstadt, Germany) and scanned for further analysis with an Olympus SLIDEVIEW VS200 slide scanner (Olympus, Hamburg, Germany) featuring a 20× objective.

### Artificial intelligence-based quantification

We used Visiopharm software version 2021.09 (Visiopharm A/S, Denmark) to develop applications for assessing the density of villi as well as number and area of vessel cross sections on villous areas of CD34 stained whole-slide images, as previously described and schematically illustrated (Zafaranieh *et al.*, 2023a). In brief, the tissue regions within the scanned samples were initially identified by applying an intensity threshold of 210 (on an 8-bit scale). Non-villous structures, such as cell islands, basal and chorionic plates, and placental septa were manually excluded from the analysis as our focus was on villi and vessels. Another thresholding application was created to pre-separate villi from background. Subsequently, an artificial intelligence (AI) application was trained with deep learning U-NET network on manually marked classes for villi, background (intervillous space), fibrin and staining artefacts on selected samples. Detected staining artefacts within the intervillous space were added to the background. In order to assess the accuracy of the classification, we applied the application (app) to the remaining set of samples. An additional app was then created to detect vessels on top of the villous area of CD34 stained sections by utilizing an eosin feature included in the Visiopharm Software to extract the CD34 stain. Several post-processing steps were carried out within the app to enhance the precision of vessel detection. This included addressing instances where vessels in proximity were not separated or where vessels were not adequately filled in earlier steps, as described in detail elsewhere (Zafaranieh *et al.*, 2023a).

Additionally, in order to reduce the impact of staining quality and non-villous structures in the analysis and to focus mostly on peripheral villi, where most vascular adaptations take place (Kingdom and Kaufmann, 1999), two to three circle regions with a diameter of 1 mm were placed on villous structures of each sample. The same steps were undertaken to quantify villous density, vessel areas and number of vessels.

**Figure 1.**
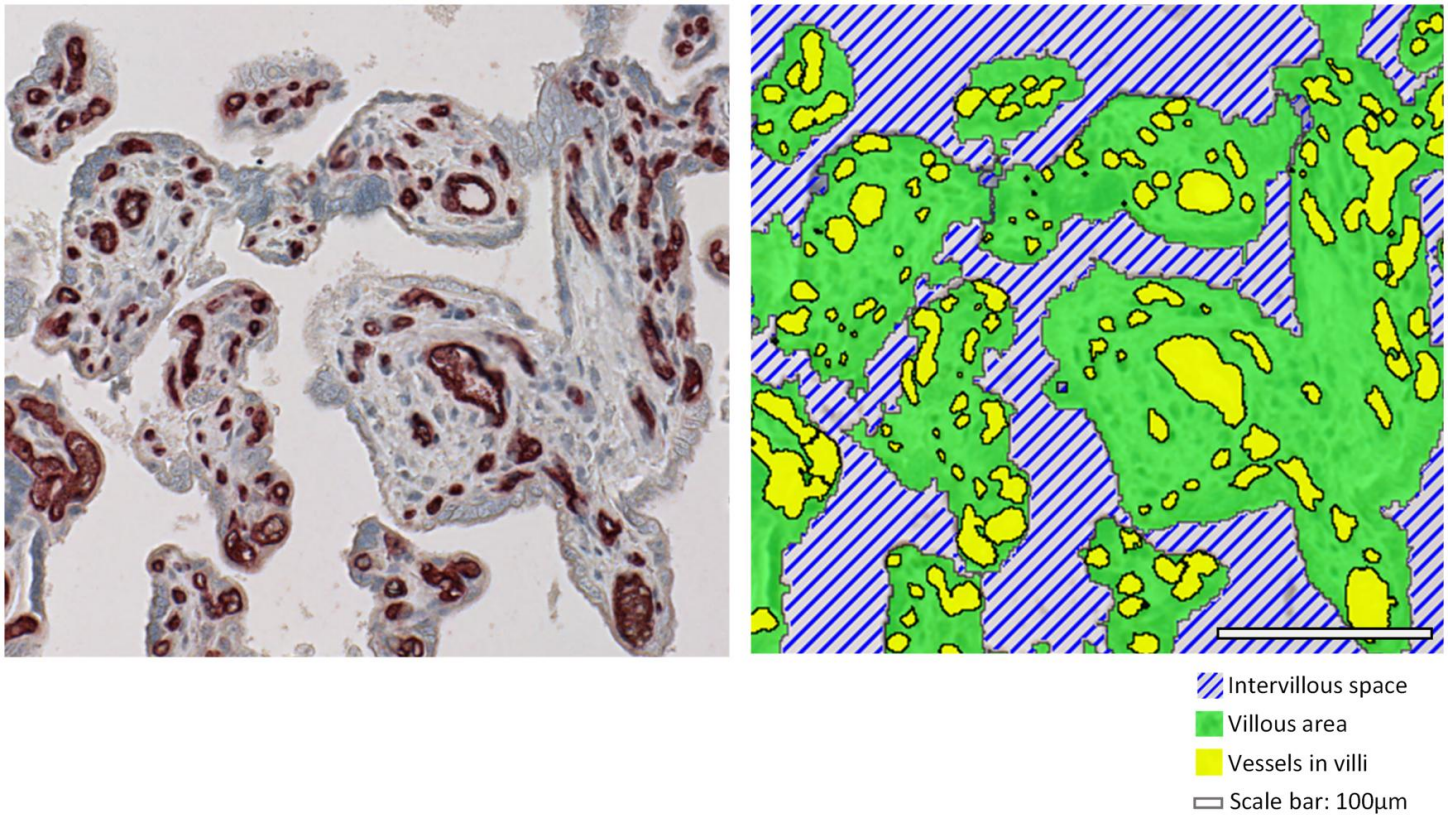
**Illustration of the AI-generated labels for villous area, intervillous space, and vessels in the human placenta**. Green: villous area, hatched blue: intervillous space, yellow: vessels. Normalized values were calculated from these parameters. % density of villi: villous surface area/(villous + intervillous area), % vessel area: vessel area/villous area, vessel count = vessel number/mm^2^ villous area.

Since image analysis runs on whole tissue sections, neither the absolute number of vessels nor the absolute villous area are useful outcomes for comparison as they depend on the size of the tissue piece in the section. Therefore, density of villi (%) was calculated as a percentage of villous area relative to total area of villi, fibrin and intervillous space. Measured numbers of vessels were normalized to 1 mm^2^ villous area (vessel count/mm^2^ villous area). Vessel area (%) was calculated as a fraction of total villous area (Fig. 1).

### mRNA isolation and quantification

After removing RNA-later from the samples, two pieces of each placenta of approximately 20 mg weight were pooled from both maternal and fetal sides. Tissue samples were homogenized using the MagNA Lyser Instrument (Roche Diagnostics, Vienna, Austria: 2–3 runs, 6500 rpm, 20 s). For RNA isolation, an miRNeasy Mini Kit (Qiagen, Hilden, Germany, #217004) was used according to the manufacturer’s protocol. RNA concentration and quality were assessed using QIAxpert (Qiagen, Germany) and Agilent 2100 Bioanalyser systems (Agilent, Santa Clara, CA, USA).

Expression levels of mRNAs for pigment epithelium-derived factor (PEDF), vascular endothelial growth factor (VEGF), and CD34 were quantified by molecular counting using NanoString nCounter Analysis Technology (Nanostring Technologies, Seattle, WA, USA). Targets were selected based on their involvement in various aspects of angiogenesis. CD34 protein expression is related to hematopoietic and vascular-associated progenitor cells. VEGF contributes to placental angiogenesis and promotes vascular expansion, whilst PEDF is an anti-angiogenic trophoblast-derived molecule that is involved in restricting the expansion of fetoplacental endothelium in late pregnancy and modulates the effects of VEGF (Vuorela *et al.*, 1997; Loegl *et al.*, 2016).

The readouts of signal intensities are given in arbitrary units (AU). Probes for targeted genes were part of a customized CodeSet (nCounter^TM^ PlexSet^TM^) comprising 24 probes, including the three validated housekeeping genes ornithine decarboxylase antizyme 1 (OAZ1), WD repeat-containing protein 45-like (WDR45L), and tata-box-binding protein (TBP). The probes were used for hybridization of a total of 490 ng RNA per sample, according to the manufacturer’s instructions. NanoString nSolver Analysis Software v4.0 (NanoString Technologies, Seattle, WA, USA) was used for quality control and normalization.

### Statistical methods

Participant characteristics are presented as mean and SD, median and Interquartile Range (IQR) or count and proportion. The characteristics of included and excluded participants were compared using the unpaired Student’s *t*-test or Chi-square test. Accelerometer data (MVPA, % ST) of different time periods were averaged and presented as mean MVPA and % ST throughout pregnancy.

Differences in placental and vascular outcomes between tertiles of mean MVPA and % ST were assessed with one-way ANOVA or Kruskal–Wallis analysis. Tukey’s Test for multiple comparisons or Bonferroni-corrected pairwise Mann–Whitney *U* test were used for post-hoc analysis. Linear regression was used to further analyze the relation between mean MVPA and % ST with morphologically assessed placental and vascular outcomes, as well as mRNA expression levels of pro- and anti-angiogenic markers. To be able to estimate associations independently of one another, MVPA and % ST variables were included in the same model. All models were adjusted for maternal age (continuous), pre-pregnancy BMI (continuous), later development of gestational diabetes (yes versus no) and fetal sex (female versus male). Adjustments for gestational age (36.1–41.9 weeks); including one preterm delivery, i.e. < 37 weeks, the mode of delivery (spontaneous versus elective), parity (nulli- versus multi-parous), smoking (yes versus no), physical activity intervention (yes versus no) and healthy eating intervention (yes versus no) did not change the associations and, therefore, were left out of the models. Effect modification by fetal sex was examined by adding interaction terms of the MVPA and ST variables and sex to each model; however, no effect modification was observed. Additionally, multilevel mixed regression models for nested data structure (clustering women in countries) were fitted to investigate the possible effects of the study centers in the different countries.

In different sensitivity analyses, smokers (n = 10), women who developed GDM (n = 31) or pre-eclampsia and/or pregnancy-induced hypertension (n = 8) were excluded from analysis.

Descriptive analysis and the main analysis of the study were conducted with IBM SPSS Statistics version 27.0.1 (IBM, Armonk, NY, USA). Plots were produced with GraphPad Prism software version 9.2.0 (GraphPad, Boston, MA, USA).

## Results

The characteristics of the study cohort are summarized in Table 1. Data were available for a total of 93 women that included accelerometer readings from at least two time periods during pregnancy comprising a minimum of three valid days of measurement per time period, placenta samples, as well as mRNA expression quantification. One outlier was removed due to poor placental staining quality resulting in 92 datasets for final analyses. The flow chart of participants and reasons of exclusion are shown in Supplementary Fig. S1. A Comparison of included and excluded women is shown in Supplementary Table S1. The majority of study participants were Caucasian, non-smoking, highly educated women, with an average age of 33.3 ± 5.4 years, and a pre-pregnancy BMI of 32.9 (IQR 4.2) kg/m^2^.

**Table 1.** Characteristics of participants in a study of placental structural adaptation to maternal physical activity and sedentary behavior.

*Maternal characteristics*	N = 92
Age, years, mean ± SD	33.3 ± 5.4
Prepregnancy BMI, kg/m^2^, median (IQR)	32.9 (4.2)
Gestational weight gain, kg, mean ± SD n = 89	8.3 ± 5.2
Nulliparous, count (%)	47 (51.1%)
High education, count (%)	55 (59.8%)
European descent, count (%)	75 (81.5%)
Smoking, count (%)	10 (10.9%)
Spontaneous delivery, count (%) n = 88	63 (71.6%)
GDM, count (%) n = 89	31 (34.8%)
PE or PIH, count (%) n = 89	8 (9.0%)

*Neonatal characteristics*	N = 92
Placenta weight, g, mean ± SD n = 88	632.3 ± 149.3
Birthweight, g, mean ± SD	3610 ± 497
Gestational age at birth, weeks, mean ± SD	40.0 ± 1.3
Female sex, count (%)	41 (44.6%)

GDM, gestational diabetes mellitus; IQR, interquartile range; PE, pre-eclampsia; PIH, pregnancy-induced hypertension.

### Physical activity and sedentary time

Women had an average daily MVPA of 39.5 (IQR 24.8) minutes and an average % ST of 72.3% ± 7.6%. Accelerometer data are presented separately for each time period in Supplementary Table S2.

### Histologically assessed placental structure

Two placental sections were stained and quantified from each woman and mean values were calculated for statistical analysis. On average, villous tissue comprised 49.7 ± 0.5% of the whole tissue in scanned images and 56.6 ± 8.9% of selected regions. The vessels comprised 15.6 ± 3.6% of the villous area in the whole section and 17.9 ± 4.6% in selected regions. In the whole section, 1324 ± 233 cross sections of vessels were counted per mm^2^ villous area. In selected regions comprising peripheral villi, the count was 1735 ± 286/mm^2^ villous area.

### Comparison of placental outcomes between MVPA and % ST tertiles

**Figure 2.**
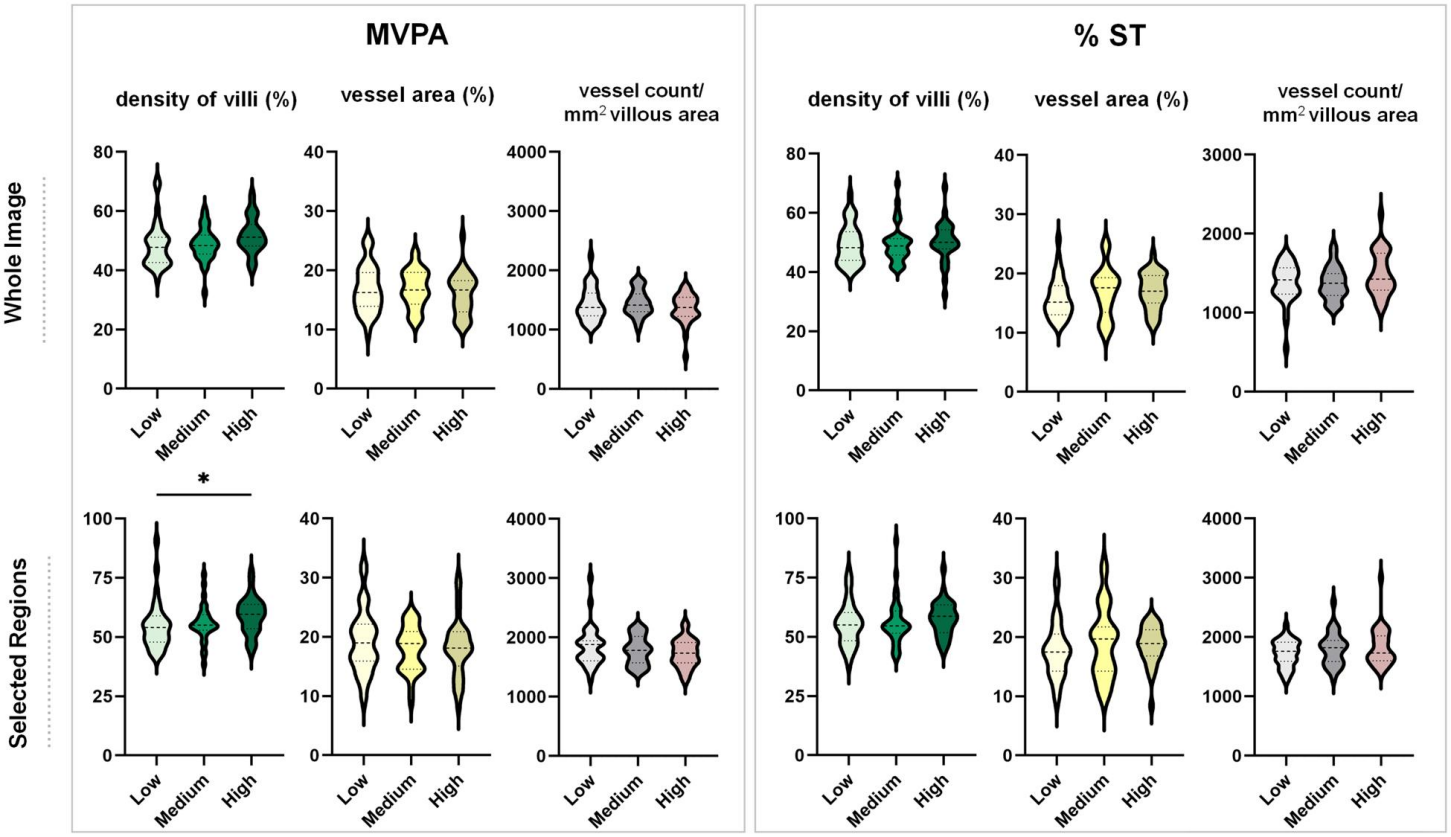
**Placental structural outcomes according to MVPA and % ST tertiles in obese pregnant women**. Results from one-way ANOVA or Kruskal–Wallis analysis. MVPA (min/day) first tertile < 30.7, second tertile 31.3–49, third tertile > 49.3; ST (%) first tertile < 69.8, second tertile 70–75.4, third tertile > 76.1. * *P* = 0.03. MVPA, moderate-to-vigorous physical activity; ST, sedentary time.

The characteristics of the study cohort in different PA and % ST tertiles are shown in Supplementary Table S3. In the placentas of women with high MVPA compared to women with low MVPA, the density of villi was higher in the whole image (median: 51.8 versus 47.8, *P* = 0.06) and selected regions (59.6 versus 54.1, *P* = 0.03), respectively. The vessel count/mm^2^ villous area and the percentage vessel area were not significantly different between different groups. No significant differences in outcomes were found between tertiles of % ST (Fig. 2).

### Association between MVPA and % ST with placental structure

In linear regression models, adjusted for maternal age, pre-pregnancy BMI and fetal sex, no associations between MVPA levels and % ST with placental weight were found (Table 2).

**Table 2.** Associations of maternal mean moderate to vigorous activity and % ST throughout pregnancy with placental structural outcomes.

	MVPA, min/day			ST, %		
	Beta (95% CI)	SB	*P*	Beta (95% CI)	SB	*P*
Placenta weight, g	−0.69 (−2.52, 1.14)	−0.09	0.46	0.45 (−4.57, 5.48)	0.02	0.86
*Whole section*						
Density of villi (%)	**0.12 (0.05, 0.2)**	**0.36**	**0.001**	**0.23 (0.04, 0.43)**	**0.26**	**0.02**
Vessel area (%)	−0.03 (−0.07, 0.01)	−0.15	0.20	0.03 (−0.08, 0.13)	0.05	0.65
Vessel count/mm^2^ villous area	0.1 (−2.86, 3.07)	0.01	0.95	4.5 (−3.19, 12.18)	0.13	0.25
*Selected regions comprising peripheral villi*						
Density of villi (%)	**0.17 (0.07, 0.26)**	**0.36**	**<0.001**	**0.35 (0.1, 0.6)**	**0.29**	**0.01**
Vessel area (%)	−0.05 (−0.1, 0.01)	−0.18	0.10	−0.03 (−0.17, 0.11)	−0.05	0.68
Vessel count/mm^2^ villous area	−1.52 (−4.68, 1.63)	−0.10	0.34	2.47 (−5.71, 10.65)	0.07	0.55

Results from linear regression models, adjusted for BMI, maternal age, gestational diabetes, and fetal sex. MVPA and % ST are simultaneously in the models, so adjusted for/independent of each other. MVPA, moderate-to-vigorous activity; SB, standardized beta; ST, sedentary time. Bold font indicates significant associations.

In whole tissue sections, MVPA level was positively associated with the density of villi (beta 0.12; 95% CI 0.05, 0.2). Interestingly, % ST was also positively related to the density of villi (0.23; CI 0.04, 0.43). Whole tissue sections comprised stem villi, mature intermediate villi, terminal villi and other placental structures such as fibrinoid (Huppertz, 2023b). To focus on villi, which predominantly contribute to the oxygen supply to the fetus, we subsequently ran the analysis on selected regions, including only peripheral villi that mostly represent terminal and mature intermediate villi. Higher mean MVPA and % ST during pregnancy were associated with increased villous density in selected regions of peripheral villi (0.17; CI 0.07, 0.26 and 0.35; CI 0.1, 0.6 respectively). The vessel count/mm^2^ villous area and % vessel area were not significantly associated with mean MVPA or % ST (Table 2).

Results of the linear regression model assessing the relations of MVPA and % ST measured at different time periods in pregnancy with placental outcomes showed that MVPA at all three time periods, including early pregnancy, was positively associated with density of villi. Higher MVPA at 35–37 weeks was associated with lower vessel area detected in selected regions, i.e. at sites with peripheral villi (−0.06; CI −0.12, −0.01). In addition, % ST at <20 and 35–37 weeks was associated with higher density of villi in selected regions (0.39; CI 0.04, 0.73) (Supplementary Table S4).

### Association of MVPA and % ST with mRNA expression of pro- and anti-angiogenic factors

In linear regression models, no significant associations of mean MVPA and % ST with mRNA expression of pro/anti-angiogenic markers in the placenta were found (Table 3).

**Table 3.** Associations of maternal mean moderate to vigorous activity and % ST throughout pregnancy with mRNA expression levels of pro and anti-angiogenic factors in the placenta.

	MVPA, min/day		ST, %	
	Beta (95% CI)	*P*	Beta (95% CI)	*P*
VEGF (AU)	0.001 (−0.01, 0.01)	0.91	0.02 (−0.01, 0.41)	0.24
PEDF (AU)	−0.002 (−0.01, 0.003)	0.35	−0.01 (−0.02, 0.01)	0.35
CD34 (AU)	−0.004 (−0.01, 0.004)	0.31	−0.01 (−0.03, 0.01)	0.44

Results from linear regression models, adjusted for BMI, maternal age, gestational diabetes, and fetal sex. MVPA and % ST are simultaneously in the models, so adjusted for/independent of each other. AU, arbitrary unit; MVPA, moderate-to-vigorous physical activity; PEDF, pigment epithelium-derived factor; ST, sedentary time; VEGF, vascular endothelial growth factor.

### Sensitivity analyses

**Figure 3.**
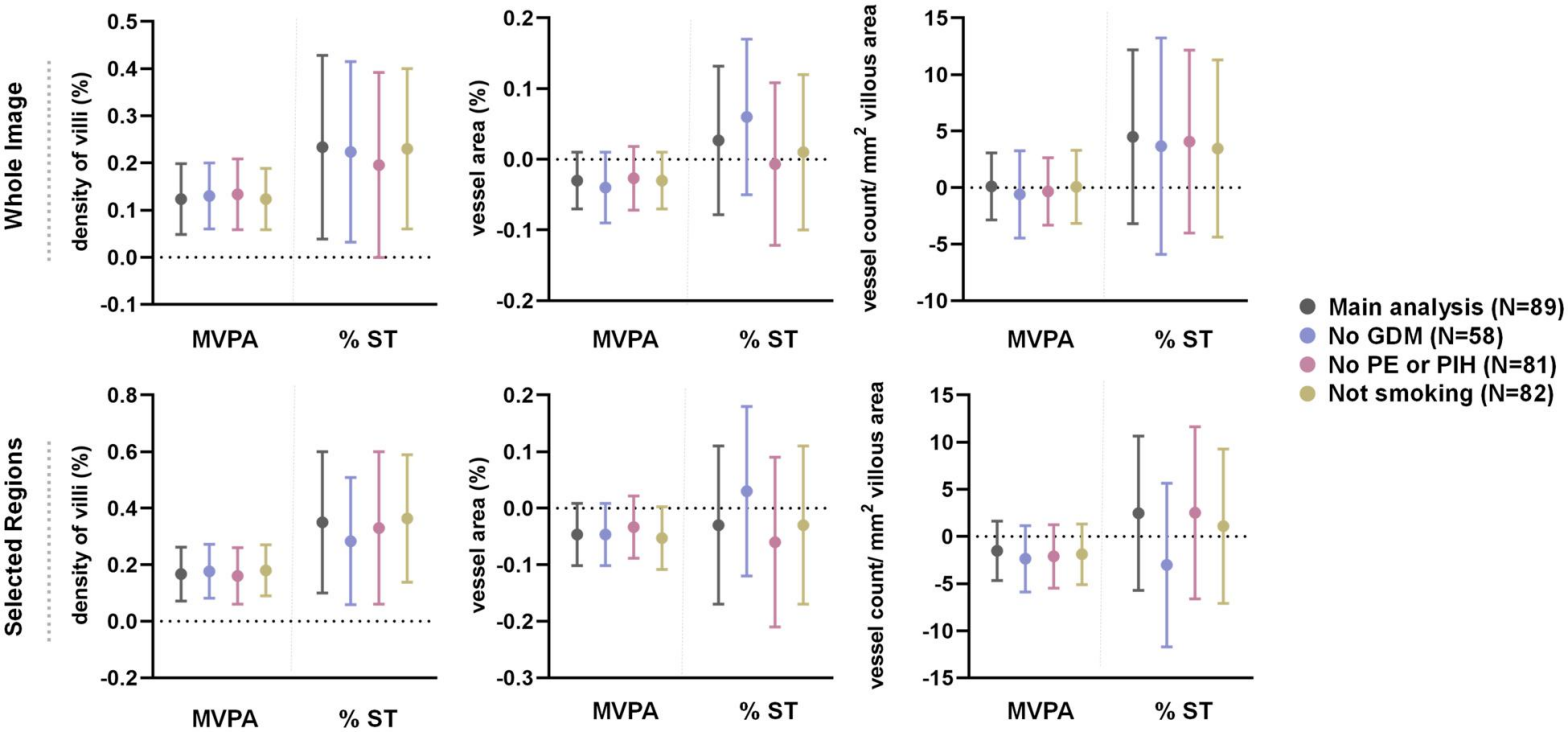
**Graphical presentation of results of sensitivity analyses showing beta coefficient estimates and 95% CI**. GDM, gestational diabetes mellitus; MVPA, moderate to vigorous activity; PE, preeclampsia; PIH, pregnancy-induced hypertension; ST, sedentary time.

The results of the main analysis and the sensitivity analyses are displayed in Fig. 3. All estimates and statistical significance of the sensitivity analyses were similar in the main analysis and the sensitivity analyses in whole image and selected regions.

Additionally, multilevel mixed regression models allowing for a random intercept for countries showed similar estimates and significance for all outcomes. Country-level variance for villous density was 3.23 (SE = 6.53) in the whole image and 5.39 (SE = 5.96) in regions. For vessel area, the country-level variance was 2.95 (SE = 2.62) in the whole image and 2.77 (SE = 2.93) in selected regions. The between-country variations were not significant. The Chi-square likelihood ratio test showed no significant improvement of a random slope model compared to the reduced model.

## Discussion

This is the first study using objectively measured PA and ST at three time periods during human pregnancy to analyze their relation with placental structures that were examined and quantified using an AI-based approach. Within our cohort of pregnant women with obesity, we found that the density of villi was higher in placentas of women who had higher average MVPA. This positive association with MVPA was found already <20 weeks and during the further course of gestation. Unexpectedly, % ST was also positively associated with the density of villi.

Villous density is a proxy measure for surface area of placental villi and is based on angiogenesis in the second half of pregnancy (Huppertz and Peeters, 2005). Diffusion-limited transfer of molecules, such as oxygen, is a function of exchange area. Hence, an increase in villous surface area may enhance oxygen uptake in a situation of potentially reduced oxygen supply as a result of reduced blood flow to the uterus. This reflects an adaptive response of the placenta and has been described also in pregnant women with iron-deficient anemia (Moeller *et al.*, 2019). Should this adaptation prove inadequate to cover fetal oxygen demand, then further mechanisms may operate including enhancing placental vascularization and vascular surface. The positive association between average maternal MVPA and density of villi may reflect such an adaptive response. This is consistent with early enlargement of ‘diffusion villi’, similar to ‘peripheral villi’ in our study, which was found in pregnant women with anemia (Moeller *et al.*, 2019). Expansion of villous surface in the second half of pregnancy depends on placental angiogenesis followed by proliferation of villous cytotrophoblasts, which subsequently fuse with the overlying syncytiotrophoblast (Huppertz, 2018). Low oxygen levels are a stimulus for proliferation of villous cytotrophoblasts and endothelial cells, a prerequisite for villous surface expansion (Huppertz *et al.*, 2003). Indeed, a greater placental proliferation index of Ki67, a commonly used proliferation marker, has been previously reported in exercising healthy pregnant women (Bergmann *et al.*, 2004). Hence, enhanced proliferation with increasing MVPA may reflect a transient oxygen deficit in the intervillous space. The positive association of MVPA at different gestational time-points (<20, 24–28, and 35–37 weeks) with density of villi at term of pregnancy suggests early onset of placental adaptation to PA of the pregnant women, which tracks throughout pregnancy. The importance of the early pregnancy period was also shown in women who started exercising early in pregnancy. In these women villous volume, and in particular that of terminal villi, which account for most of the oxygen transfer, was increased (Clapp *et al.*, 2000a).

This notion is supported by a previous study, which found higher and persistent placental growth in women who went through high-intensity exercise in early gestation compared to those starting with lower intensity (Clapp *et al.*, 2002; Kubler *et al.*, 2022). This may argue for an exercise intensity-dependent placental growth at different time-points. Moreover, ultrasonographic assessments showed a higher placental volume in women who maintained a regular exercise regimen throughout the second trimester (Clapp and Rizk, 1992). These data are consistent with our interpretation and support the concept of placental adaptations to maternal exercise. The increase in villous density and, therefore, surface area improves the ability of the villous tissue to transfer oxygen from maternal to fetal blood.

The positive relation between % ST and density of villi was unexpected. Although we do not know how the pregnant women spent their ST, these results may also be interpreted as adaptation to a postulated transient oxygen deficiency. Excessive time spent in a sitting position reduces blood flow to peripheral organs (Padilla and Fadel, 2017), which may also include the pregnant uterus, but this awaits independent demonstration.

We did not find a statistically significant relation between MVPA and the area and number of vessels. Placental vascularization is plastic and responds to local as well as fetal oxygen concentrations. In a situation of a fetal oxygen deficit, as may accompany pregnancies in women with obesity, the degree of vascularization is increased. This is a strategy to adapt to fetal demands (Desoye and Carter, 2022). Vasculogenesis and pro-angiogenic factors, such as VEGF and fibroblast growth factor, are induced by low oxygen levels (Fong, 2009). Unchanged relative vessel number and area of vessels with increasing MVPA suggests that the increase in villous surface area was sufficient to supply the fetus with adequate oxygen. In this context, it is essential to highlight that the absence of distinctions in the number and area of vessels, which have been standardized relative to the villi area, in women with different levels of MVPA does not conclusively infer that vascularization did not either precede or succeed the expansion of villi. Rather, it signifies the absence of notable vascular expansion (i.e. hypervascularization) concomitant with higher MVPA. The fetus may have mounted adaptive responses itself (Clapp *et al.*, 1993; Desoye and Carter, 2022); however, a respective analysis was outside the aim of the present study.

Our results differ from another study, which found an increase in placental absolute (total villous vascular volume) and relative (total villous vascular volume/total villous volume) villous vascular volume in women who followed a regular running regimen during pregnancy (Bergmann *et al.*, 2004). These discrepancies may be accounted for by different PA intensities and study population, as the women in our study were overweight/obese. Another reason may be the different techniques used to count villous structures. Bergmann *et al.* (2004) used three images (×200 magnification) per case to count structures using a point grid with 150 dots. Thus, these authors counted 450 structures in total, including villi, non-villous areas and vessels. In our approach, we evaluated full sections including an average of 41.3 mm^2^ tissue surface area and 29 393 vessels per section.

**Figure 4.**
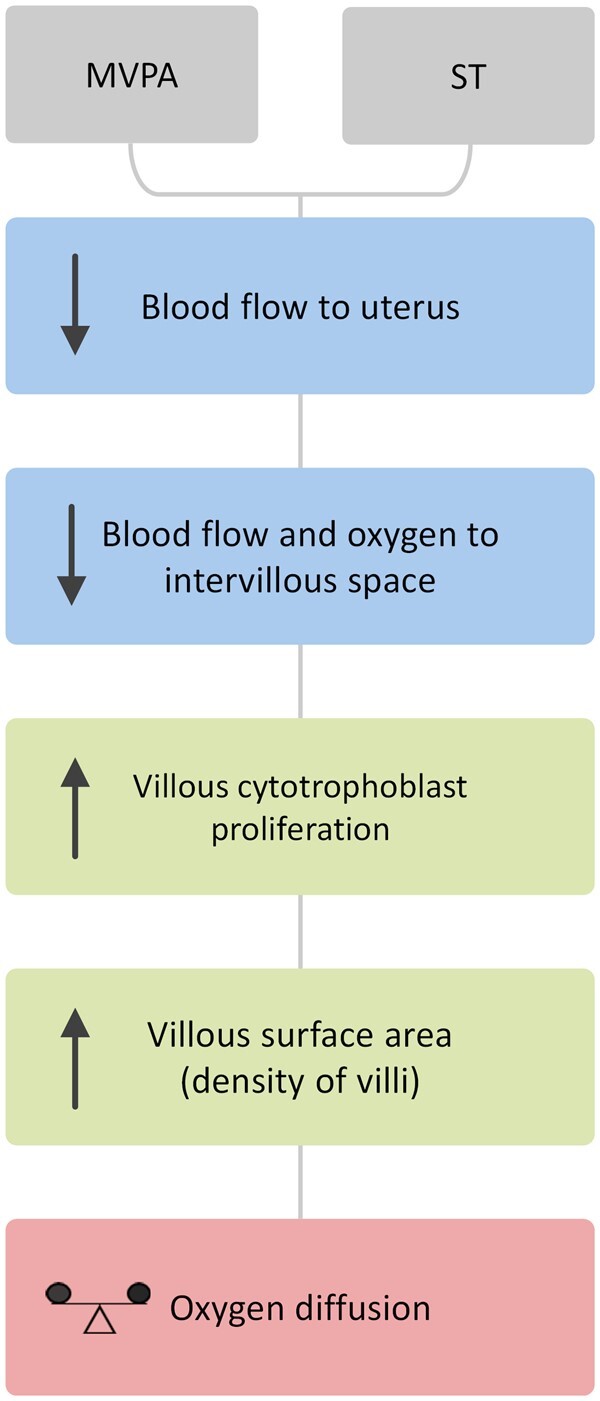
**Proposed schematic of the structural adaptation of the human placenta to higher levels of MVPA and ST**. Both MVPA and ST lead to decreased blood flow to the uterus and, consequently, an oxygen deficit in the intervillous space. The placenta adapts its structure to the intermittent oxygen deficit by increasing villous density, a proxy measure of placental surface area. This adaptation increases oxygen transmission from the maternal to the fetal circulation, thus resulting in a stable oxygen supply to the fetus. MVPA, moderate-to-vigorous physical activity; ST, sedentary time.

Placental expression of pro- and anti-angiogenic factors did not associate with mean MVPA or % ST in our cohort of obese pregnant women. In a previous study comparing active and non-active pregnant women, mRNA and protein levels of VEGF and its receptor VEGFR-1 were higher in placentas of women who were following or exceeding PA recommendations in pregnancy, i.e. 150 min of moderate PA per week, compared to non-active pregnant women (Bhattacharjee *et al.*, 2021). The disparity might be due to the small MVPA range in our study as the majority of the women were meeting these recommendations. In addition, higher PA intensities could further promote the vascular network in the placenta, which is accompanied by an expansion of villous structures. As a speculation, during maternal PA the blood supply to skeletal muscles and heart of the mother is prioritized, reducing uterine arterial blood supply to the placenta (Clapp *et al.*, 2000b). As a result of MVPA, oxygen tension in the intervillous space is likely to drop intermittently and transiently. This appears to elicit a molecular response that increases placental surface area and may contribute to oxygen transfer from maternal blood to an extent that is sufficient to cover fetal oxygen demand (Desoye and Carter, 2022). Hence, an additional increase in placental vascularization is not needed as a further adaptation. In general, an expansion of placental exchange area as result of an increase in surface area of functional villous structures and/or placental blood vessels should be associated with enhanced placental oxygen supply to the fetus (Fig. 4).

### Strengths and weaknesses

The objective PA measurements obviate the need for self-reports, as these poorly correlate with objective measures (Oostdam *et al.*, 2013). The repeated PA and ST measurements throughout the course of pregnancy offer a better representation of the volume and intensity of PA than a single time-point measurement. These measurements also enabled us to assess potential time-dependent PA effects on outcomes. AI stereological image analysis allows for the examination of a greater number of samples, which increases consistency and reduces bias as compared to conventional observer-dependent assessments. Expanding the study’s sample size, indicated by an increase in the number of included placentas, is an effective strategy for mitigating the impact of sampling variation. Furthermore, AI-based analysis enables the assessment of staining on entire sections. In our case, with an average section size of 0.4 cm^2^, this scale is approximately more than 100 times larger than a single field of view examined and quantified under a microscope. Hence, this captures more of the tissue heterogeneity compared to conventional methods.

Some weaknesses have to be acknowledged. The outcome measurements are proxies for the surface areas and the number of vessels; however, the arrangement and the distance of the vessels from the villous borders, the rate of the blood flow, and the diffusion properties of the gas could also affect the efficiency of oxygen delivery, occurring, or not, as a short-term response (Carter, 2009). The analysis of samples derived from human studies provides a glimpse into the state of the term placenta when the mRNA expression and morphological assessments were performed. Nevertheless, the placenta undergoes dynamic changes throughout the course of gestation, and these placental adaptations play a pivotal role in determining the outcomes of pregnancy. We did not use conventional stereological methods to quantify morphometric outcomes as the required tissue sampling strategies were not feasible in the setting of our multicentric randomized clinical trial, yet tissue embedding followed a random orientation strategy and we used a stereology software. Moreover, tissue sampling, the fixation approach, and duration as well as storage of the samples might induce variations (Kozai *et al.*, 2024). However, tissue collection followed strict protocols and research nurses of the individual study sites received standardized training for the procedures. The study collective comprised mostly Caucasian women with a BMI ≥ 29 kg/m^2^, the majority of whom were meeting PA guidelines for pregnant women. Future studies in other cohorts with GDM, fetal growth restriction, lean women, or those with different ethnicities or varying ranges of PA levels will be required to assess whether the results can be generalized. In addition, we included only a subgroup of DALI lifestyle trial participants in this study. Nonetheless, except for maternal age, delivery mode, and birthweight, the subgroup is representative of the total study population (Supplementary Table S1). Given that accelerometers cannot specify the type of PA or exercise, it is imperative to explore this aspect in future research with the view to translate the results into lifestyle guidance for pregnant women with obesity.

## Conclusion

Both PA and ST in obese pregnant women are associated with higher villous density, a proxy measure for the placental surface towards maternal blood. The increase in villous density and, therefore, surface area may have occurred already from early on in pregnancy and improves the ability of the villous tissue to transfer oxygen from maternal to fetal blood. The association of ST with increased villi density is unexpected and deserves further investigation.

## Supplementary Material

deae090_Supplementary_Figure_S1

deae090_Supplementary_Table_S1

deae090_Supplementary_Table_S2

deae090_Supplementary_Table_S3

deae090_Supplementary_Table_S4

## Data Availability

In adherence to ethical regulations, the raw data cannot be accessed online. However, the data supporting the conclusions of this manuscript will be made available by the authors, on request to the corresponding author.
