# Correction: Placental structural adaptation to maternal physical activity and sedentary behavior: findings of the DALI lifestyle study

**DOI:** 10.1093/humrep/deae173

**Published:** 2024-07-29

This is a correction to: Saghi Zafaranieh, Monika Siwetz, Barbara Leopold-Posch, Daniel Kummer, Berthold Huppertz, Gernot Desoye, Mireille van Poppel, DALI Core Investigator Group, Placental structural adaptation to maternal physical activity and sedentary behavior: findings of the DALI lifestyle study, *Human Reproduction*, Volume 39, Issue 7, July 2024, Pages 1449–1459, https://doi.org/10.1093/humrep/deae090

In the originally published version of the manuscript there was a typographical error in one of the names of the collaborator Group, DALI Core investigator Group. This should read: “Ewa Wender-Ozegowska” instead of: “Ewa Wender-Oegowska”.

The emendation has been made to the article.

