# Supplementary material for: Placental structural adaptation to maternal physical activity and sedentary behavior: findings of the DALI lifestyle study
Source: Hum Reprod. 2024 May 10;39(7):1449–59. doi: 10.1093/humrep/deae090 (PMC11776022; doi:10.1093/humrep/deae090)
Supplement: deae090_Supplementary_Figure_S1 [file deae090_supplementary_figure_s1.pdf]

OXFORD

human reproduction

Human Reproduction, 2024, 1–1
https://doi.org/10.1093/humrep/deae090
Supplementary Data

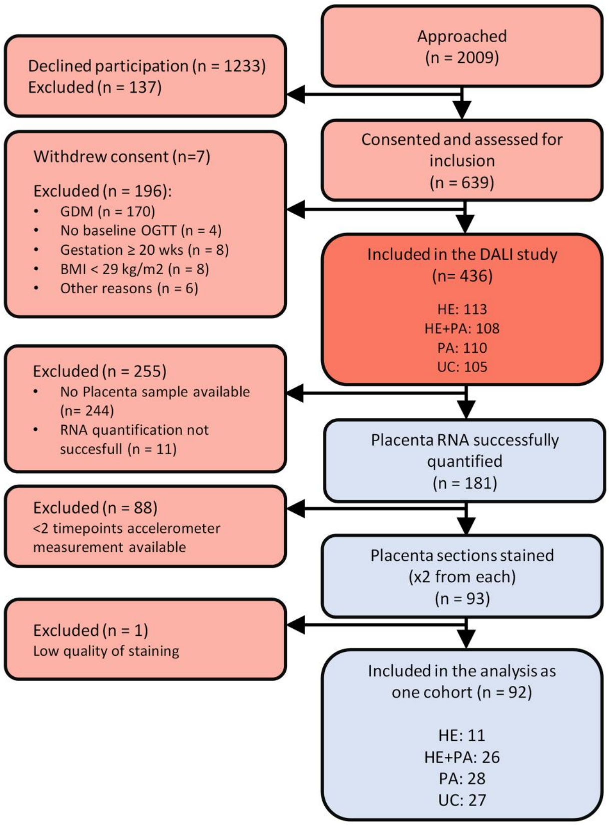

Supplementary Figure S1. Flow diagram of study participants. GDM, gestational diabetes mellitus; HE, healthy eating; OGTT, oral glucose tolerance test; PA, physical activity; UC, under control.
