# Supplementary material for: Placental structural adaptation to maternal physical activity and sedentary behavior: findings of the DALI lifestyle study
Source: Hum Reprod. 2024 May 10;39(7):1449–59. doi: 10.1093/humrep/deae090 (PMC11776022; doi:10.1093/humrep/deae090)
Supplement: deae090_Supplementary_Table_S1 [file deae090_supplementary_table_s1.pdf]

OXFORD human reproduction

Human Reproduction, 2024, 1–1
https://doi.org/10.1093/humrep/deae090
Supplementary Data

Supplementary Table S1. Characteristics of women selected for analyses and those excluded.

Maternal characteristics	Included N = 92	Excluded N = 493	*P*
Age, years, mean ± SD	**33.3 ± 5.4**	**31.6 ± 5.4**	**0.01**
Prepregnancy BMI, kg/m^2, median (IQR)	32.9 (4.2)	32.9 (5.5)	0.88
Gestational weight gain, kg, mean ± SD, n = 469	8.3 ± 5.2	7.9 ± 4.6	0.48
Nulliparous, count (%)	47 (51.1%)	250 (50.7%)	0.95
High education, count (%)	55 (59.8%)	263 (53.5%)	0.26
European descent, count (%)	75 (81.5%)	433 (88%)	0.10
Smoking, count (%) n = 548	10 (10.9%)	68 (15.1%)	0.29
Spontaneous delivery, count (%) n = 508	**63 (71.6%)**	**249 (59.3%)**	**0.03**
GDM, count (%) n = 476	31 (34.8%)	132 (34.1%)	0.90
Neonatal characteristics			
Birthweight n = 520	**3610 ± 497**	**3448 ± 562**	**0.01**
Gestational age at birth n = 527	40.0 ± 1.3	39.5 ± 4.3	0.34
Female sex n = 529	41 (44.6%)	217 (49.7%)	0.38

GDM, gestational diabetes mellitus; IQR: interquartile range. Bold font indicates significant difference between included and excluded participants.
