# Supplementary material for: Placental structural adaptation to maternal physical activity and sedentary behavior: findings of the DALI lifestyle study
Source: Hum Reprod. 2024 May 10;39(7):1449–59. doi: 10.1093/humrep/deae090 (PMC11776022; doi:10.1093/humrep/deae090)
Supplement: deae090_Supplementary_Table_S2 [file deae090_supplementary_table_s2.pdf]

human reproduction

Human Reproduction, 2024, 1–1
https://doi.org/10.1093/humrep/deae090
Supplementary Data

Supplementary Table S2. Moderate-to-vigorous physical activity and ST at three different time points in pregnancy.

	<20 weeks n = 83	24-–28 weeks n = 81	35-–37 weeks n = 72
MVPA, min/day, median (IQR)	42.7 (30.2)	39.0 (30.9)	31.7 (25.2)*
ST, % of wear time, mean ± SD	71.5 ± 9.2	73.8 ± 10.3*	75.1 ± 10.2*

* $P < 0.05$ compared to <20 weeks.
MVPA, moderate-to-vigorous physical activity; IQR, interquartile range.
