# Supplementary material for: Placental structural adaptation to maternal physical activity and sedentary behavior: findings of the DALI lifestyle study
Source: Hum Reprod. 2024 May 10;39(7):1449–59. doi: 10.1093/humrep/deae090 (PMC11776022; doi:10.1093/humrep/deae090)
Supplement: deae090_Supplementary_Table_S3 [file deae090_supplementary_table_s3.pdf]

OXFORD
human reproduction
Human Reproduction, 2024, 1–1
https://doi.org/10.1093/humrep/deae090
Supplementary Data

Supplementary Table S3. Characteristics of the included participants between different PA and ST% tertile.

	All included N = 92	MVPA First tertile N = 31	MVPA Second tertile N = 30	MVPA Third tertile N = 31	ST First tertile N = 31	ST Second tertile N = 30	ST Third tertile N = 31
Maternal PA & ST%							
MVPA, min/day, median (IQR)	39.5 (24.8)	22.30 (9.2)	39.50 (10.3)[¥]	55.3 (18.3)[¥¥]	47.8 (24.1)	45.0 (25.1)*	31.9 (28.1)**
ST, % of wear time, mean ± SD	72.1 ± 7.6	74.9 ± 9.8	72.1 ± 6.5	70.5 ± 5.7	64.6 ± 5.5	72.7 ± 1.4	80.0 ± 3.0*
Maternal characteristics							
Age, years, mean ± SD	33.3 ± 5.4	34.4 ± 5.9	33.3 ± 5.4	33.3 ± 5.4	34.2 ± 4.1	32.9 ± 6.3	33.1 ± 6.0
Prepregnancy BMI, kg/m^2, median (IQR)	32.9 (4.2)	33.1 (4.3)	32.9 (6.8)	32.1 (4.0)	32.2 (2.7)	33.8 (6.5)	32.9 (4.0)
Gestational weight gain, kg, mean ± SD n = 89	8.3 ± 5.2	8.6 ± 4.2	8.3 ± 6.1	7.8 ± 5.3	8.9 ± 5.3	7.0 ± 5.3	8.9 ± 5.3
Nulliparous, count (%)	47 (51.1%)	15 (48.4%)	17 (56.7%)	15 (48.4%)	8 (25.8%)	14 (46.7%)	25 (80.6%)**
High education, count (%)	55 (59.8%)	18 (58.1%)	16 (53.3%)	21 (67.7%)	16 (51.6%)	20 (66.7%)	19 (61.3%)
European descent, count (%)	75 (81.5%)	28 (90.3%)	26 (86.7%)	21 (67.7%)	25 (80.6%)	24 (80%)	26 (83.9%)
Smoking, count (%)	10 (10.9%)	3 (9.7%)	2 (6.7%)	5 (16.1%)	1 (3.2%)	6 (20%)	3 (9.7%)
Spontaneous delivery, count (%) n = 88	63 (71.6%)	20 (71.4%)	21 (72.4%)	22 (71%)	20 (64.5%)	21 (75%)	22 (75.9%)
GDM, count (%) n = 89	31 (34.8%)	8 (27.6%)	10 (33.3%)	13 (43.4%)	8 (26.7%)	12 (41.4%)	11 (36.7%)
PE or PIH, count (%) n = 89	8 (9.0%)	3 (10.3%)	3 (10.3%)	2 (6.5%)	3 (10%)	1 (3.4%)	4 (13.3%)
Neonatal characteristics							
Placenta weight, g, mean ± SD n = 88	632.3 ± 149.3	648.7 ± 122	656.64 ± 140	597.9 ± 172.8	613.9 ± 121.0	667.1 ± 168.8	619.3 ± 155.0
Birthweight, g, mean ± SD	3610 ± 497	3654.4 ± 374.4	3711.7 ± 500.5	3492.7 ± 569.4	3637.2 ± 489.8	3653.2 ± 525.6	3512.5 ± 484.1
Gestational age at birth, weeks, mean ± SD	40.0 ± 1.3	40.0 ± 1.3	39.8 ± 1.4	40.0 ± 1.4	39.8 ± 1.3	40.0 ± 1.2	40.1 ± 1.3
Female sex, count (%)	41 (44.6%)	14 (45.2%)	10 (33.3%)	17 (54.8%)	12 (38.7%)	14 (46.7%)	15 (48.4%)

GDM, gestational diabetes mellitus; IQR, interquartile range; MVPA, moderate-to-vigorous physical activity; PA, physical activity; PE, pre-eclampsia, PIH, pregnancy-induced hypertension; ST, sedentary time.

[¥] $P < 0.05$ compared to MVPA first tertile.
[¥¥] $P < 0.05$ compared to MVPA first and second tertile.
* $P < 0.05$ compared to ST% first tertile.
** $P < 0.05$ compared to ST% first and second tertile.
