# Supplementary material for: Placental structural adaptation to maternal physical activity and sedentary behavior: findings of the DALI lifestyle study
Source: Hum Reprod. 2024 May 10;39(7):1449–59. doi: 10.1093/humrep/deae090 (PMC11776022; doi:10.1093/humrep/deae090)
Supplement: deae090_Supplementary_Table_S4 [file deae090_supplementary_table_s4.pdf]

Human Reproduction, 2024, 1–1
https://doi.org/10.1093/humrep/deae090
Supplementary Data

Supplementary Table S4. Associations of maternal physical activity and ST at three different time points in pregnancy with placental structural outcomes.

		<20 weeks N = 80 Beta (95% CI)	SB	P	24–28 weeks N = 78 Beta (95% CI)	SB	P	35–37 weeks N = 72 Beta (95% CI)	SB	P
Placenta weight, g	MVPA, min/day	−0.37 (−1.97, 1.22)	−0.06	0.64	−1.22 (−2.60, 0.16)	−0.21	0.08	0.32 (−1.87, 1.93)	0.01	0.97
	ST	2.72 (−1.68, 7.12)	0.17	0.22	−1.62 (−6.25, 3.01)	−0.09	0.49	−1.98 (−8.46, 4.50)	−0.09	0.54
Whole-image										
Density of villi (%)	MVPA, min/day	**0.08 (0.01, 0.14)**	**0.28**	**0.03**	**0.07 (0.01, 0.13)**	**0.27**	**0.02**	**0.12 (0.04, 0.21)**	**0.39**	**0.01**
	ST, %	**0.22 (0.04, 0.40)**	**0.30**	**0.02**	0.18 (−0.01, 0.37)	0.21	0.07	0.24 (−0.03, 0.53)	0.25	0.08
Vessel area (%)	MVPA, min/day	−0.02 (−0.05, 0.02)	−0.10	0.44	−0.02 (−0.06, 0.01)	−0.17	0.15	−0.02 (−0.07, 0.03)	−0.12	0.41
	ST, %	0.06 (−0.05, 0.16)	0.15	0.28	0.03 (−0.08, 0.13)	0.06	0.61	−0.03 (−0.18, 0.12)	−0.06	0.67
Vessel count/mm^2 villous area	MVPA, min/day	−0.06 (−2.73, 2.60)	−0.01	0.96	−0.71 (−3.11, 1.68)	−0.07	0.56	2.03 (−1.25, 5.31)	0.17	0.22
	ST, %	7.11 (−0.01, 14.23)	0.25	0.05	2.43 (−5.27, 10.13)	0.08	0.53	5.51 (−5.27, 16.28)	0.15	0.30
Selected regions comprising peripheral villi										
Density of villi (%)	MVPA, min/day	**0.11 (0.02, 0.20)**	**0.30**	**0.02**	0.07 (−0.04, 0.15)	0.21	0.06	**0.17 (0.07, 0.28)**	**0.43**	**0.002**
	ST, %	**0.33 (0.09, 0.57)**	**0.34**	**0.01**	0.23 (−0.02, 0.48)	0.21	0.07	**0.39 (0.04, 0.73)**	**0.29**	**0.03**
Vessel area (%)	MVPA, min/day	−0.01 (−0.07, 0.04)	−0.06	0.61	−0.03 (−0.07, 0.01)	−0.17	0.14	**−0.06 (−0.12, −0.01)**	**−0.30**	**0.03**
	ST, %	0.01 (−0.15, 0.13)	−0.01	0.92	0.02 (−0.12, 0.16)	0.30	0.76	−0.14 (−0.33, 0.05)	−0.20	0.14
Vessel count/mm^2 villous area	MVPA, min/day	−1.18 (−4.02, 1.65)	−0.10	0.41	−1.26 (−3.82, 1.30)	−0.11	0.33	−0.69 (−4.24, 2.86)	−0.05	0.70
	ST, %	6.41 (−1.17, 14.00)	0.21	0.10	2.03 (−6.19, 10.25)	0.06	0.62	−0.10 (−11.76, 11.55)	−0.00	0.99

Linear regression models, adjusted for BMI, maternal age, gestational diabetes, and fetal sex. MVPA and % ST time are simultaneously in the models, so adjusted for/independent of each other. SB: Standardized Beta, MVPA: moderate-to-vigorous physical activity; ST, sedentary time. Significant results are presented in bold.
